# Supplementary material for: Antibacterial Metabolites Produced by Limonium lopadusanum, an Endemic Plant of Lampedusa Island
Source: Biomolecules. 2024 Jan 22;14(1):134. doi: 10.3390/biom14010134 (PMC10813400; doi:10.3390/biom14010134)

SUPPORTING MATERIAL

Antibacterial metabolites produced by *Limonium lopadusanum*, an endemic plant of Lampedusa island

Ernesto Gargiulo[1,†], Emanuela Roscetto[2,†], Umberto Galdiero[2], Giuseppe Surico[3], Maria Rosaria Catania[2], Antonio Evidente[4] and Orazio Taglialatela-Scafati[1,*]

1 Department of Pharmacy, University of Naples Federico II, Via Domenico Montesano, 49, 80131 Napoli, Italy

2 Department of Molecular Medicine and Medical Biotechnologies, University of Naples Federico II, Via Pansini 5, 80131 Napoli, Italy
3 Department of Agriculture, Food, Environment, and Forestry (DAGRI), Section of Agricultural Microbiology, Plant Pathology and Enthomology, University of Florence, 50121 Firenze, Italy
4 Institute of Sciences of Food Production, National Research Council, Via Amendola 122/O, 70125 Bari, Italy

* Correspondence: scatagli@unina.it (O.T.S.); Tel.: (+39 081678509)
† These Authors contributed equally

Summary

Figure S1. ^{1}H NMR spectrum of compound **1** (700 MHz) in $CDCl_3$ 3

Figure S2. ^{1}H NMR spectrum of compound **2** (600 MHz) in $CDCl_3$ 3

Figure S3. ^{1}H NMR spectrum of compound **3** (600 MHz) in $CDCl_3$ 4

Figure S4. ^{1}H NMR spectrum of compound **4** (700 MHz) in $CDCl_3$ 4

Figure S5. COSY 2D NMR spectrum of compound **4** (700 MHz) in $CDCl_3$ 5

Figure S6. HSQC 2D NMR spectrum of compound **4** (700 MHz) in $CDCl_3$ 5

Figure S7. HMBC 2D NMR spectrum of compound **4** (700 MHz) in $CDCl_3$ 6

Figure S8. ^{1}H NMR spectrum of compound **5** (600 MHz) in $CDCl_3$ 6

Figure S1. ^{1}H NMR spectrum of compound **1** (700 MHz) in $CDCl_3$

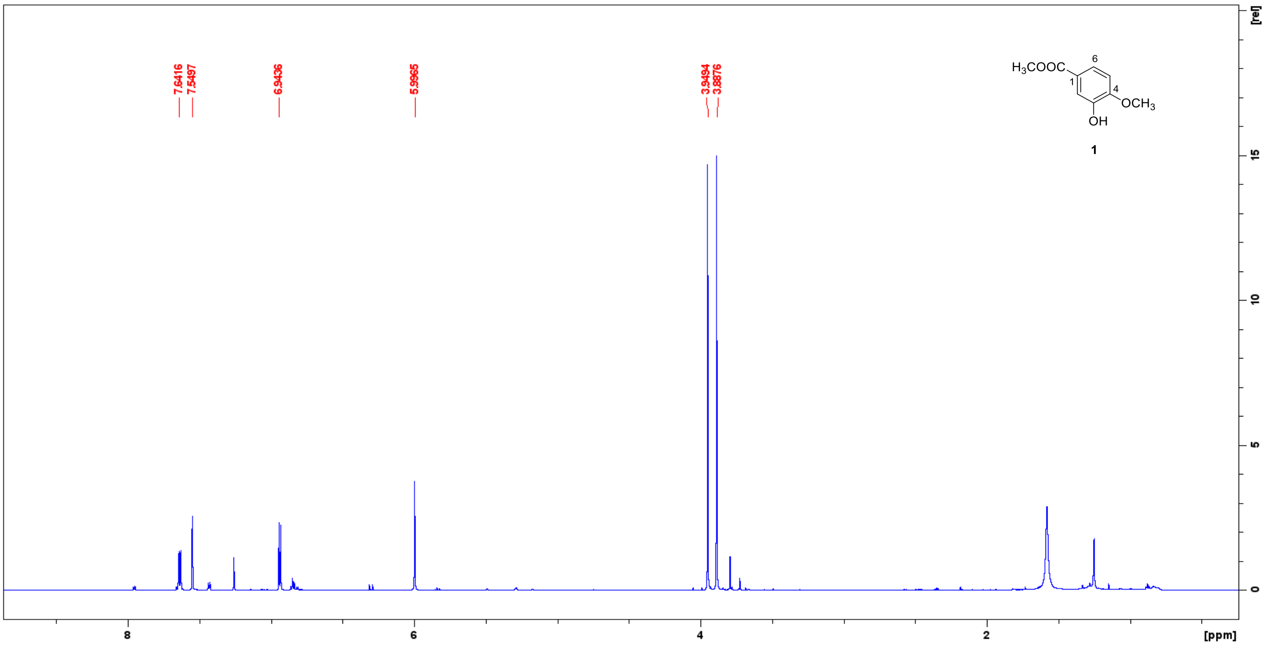

Figure S2. ^{1}H NMR spectrum of compound **2** (600 MHz) in $CDCl_3$

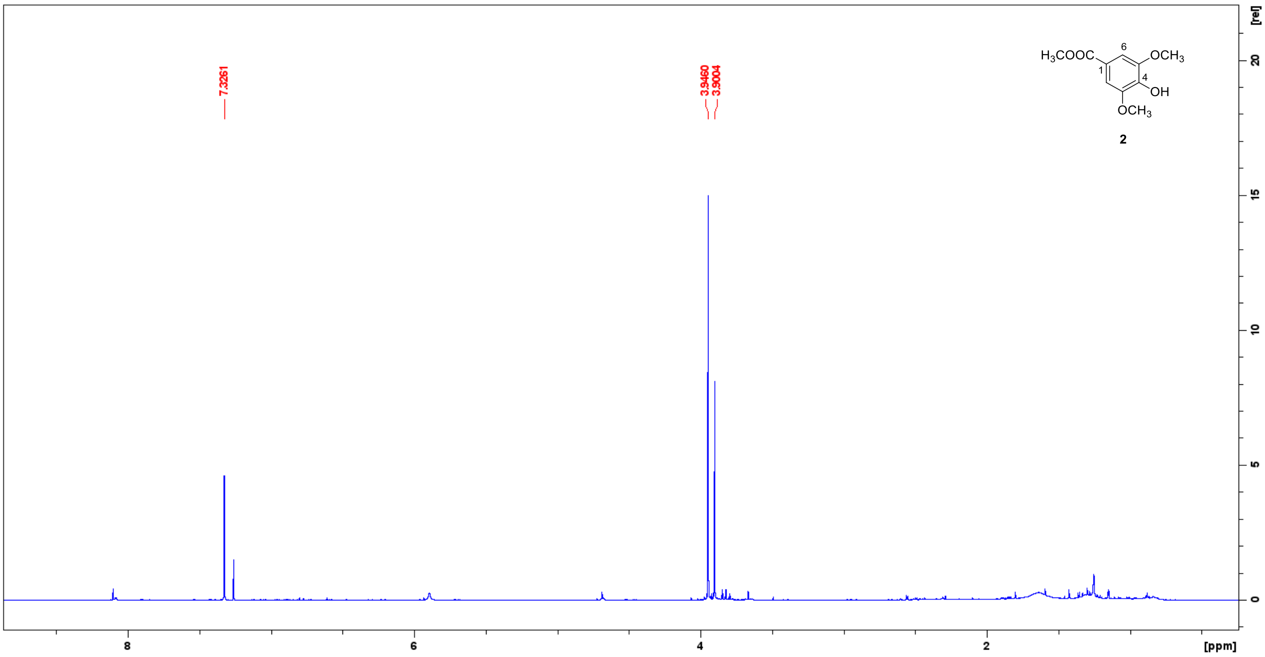

Figure S3. ^{1}H NMR spectrum of compound **3** (600 MHz) in $CDCl_3$

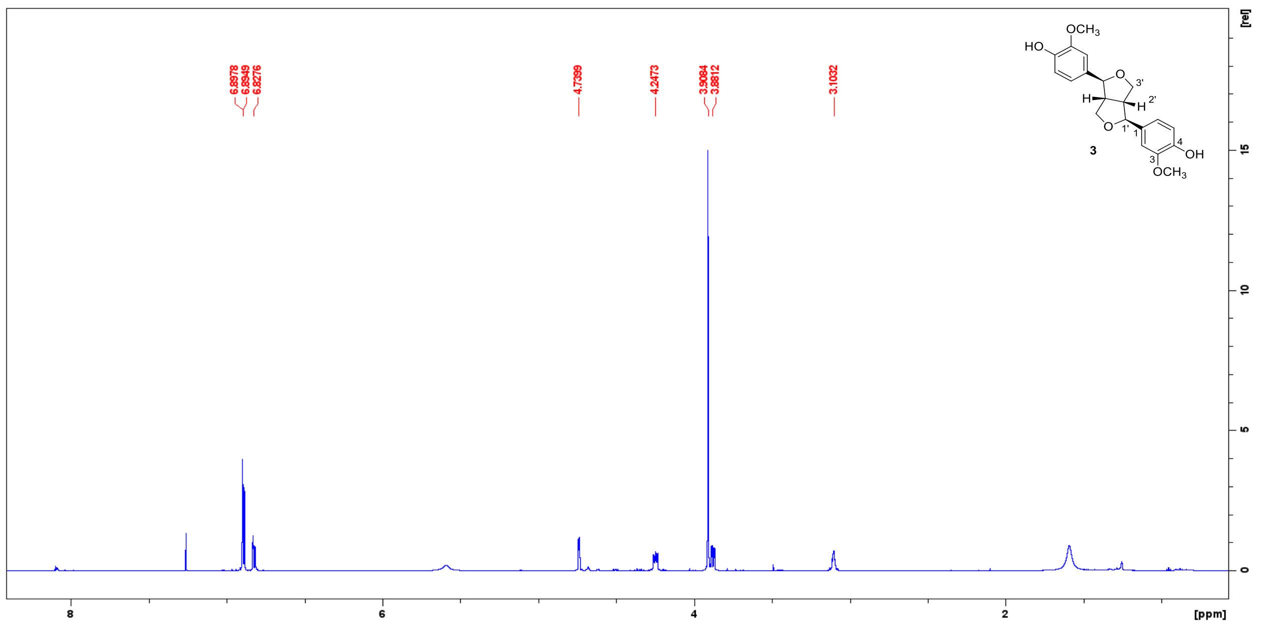

Figure S4. ^{1}H NMR spectrum of compound **4** (700 MHz) in $CDCl_3$

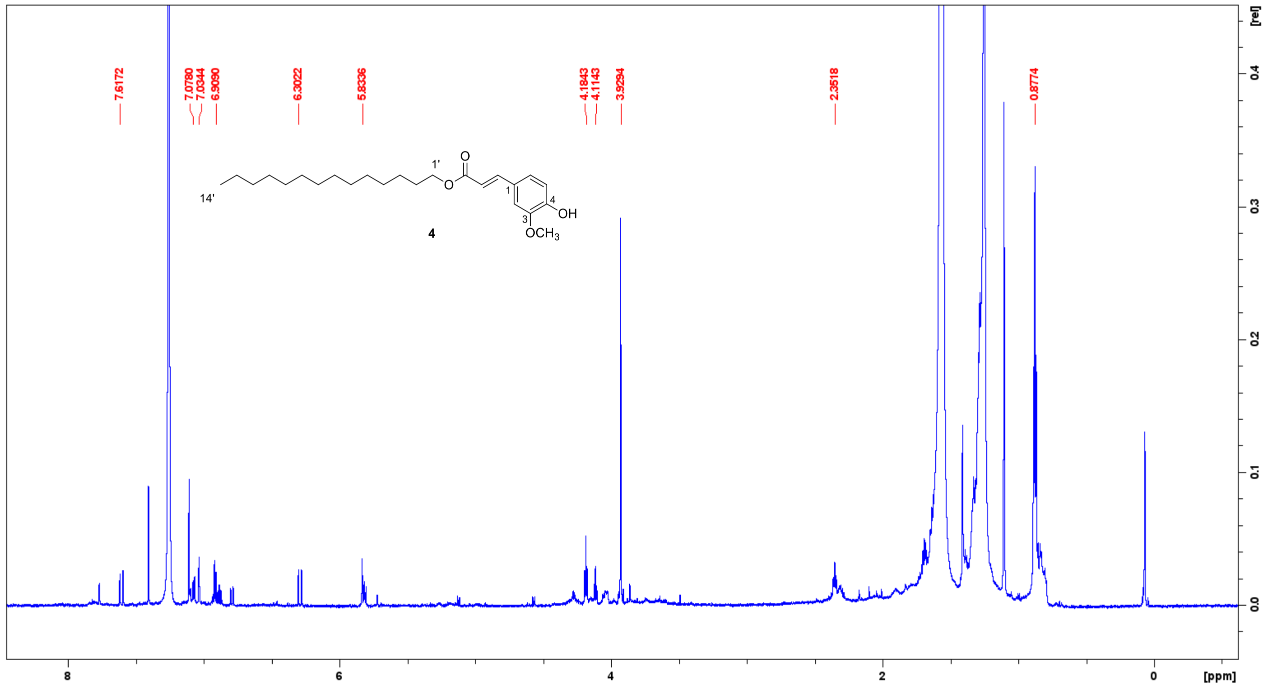

Figure S5. COSY 2D NMR spectrum of compound **4** (700 MHz) in $CDCl_3$

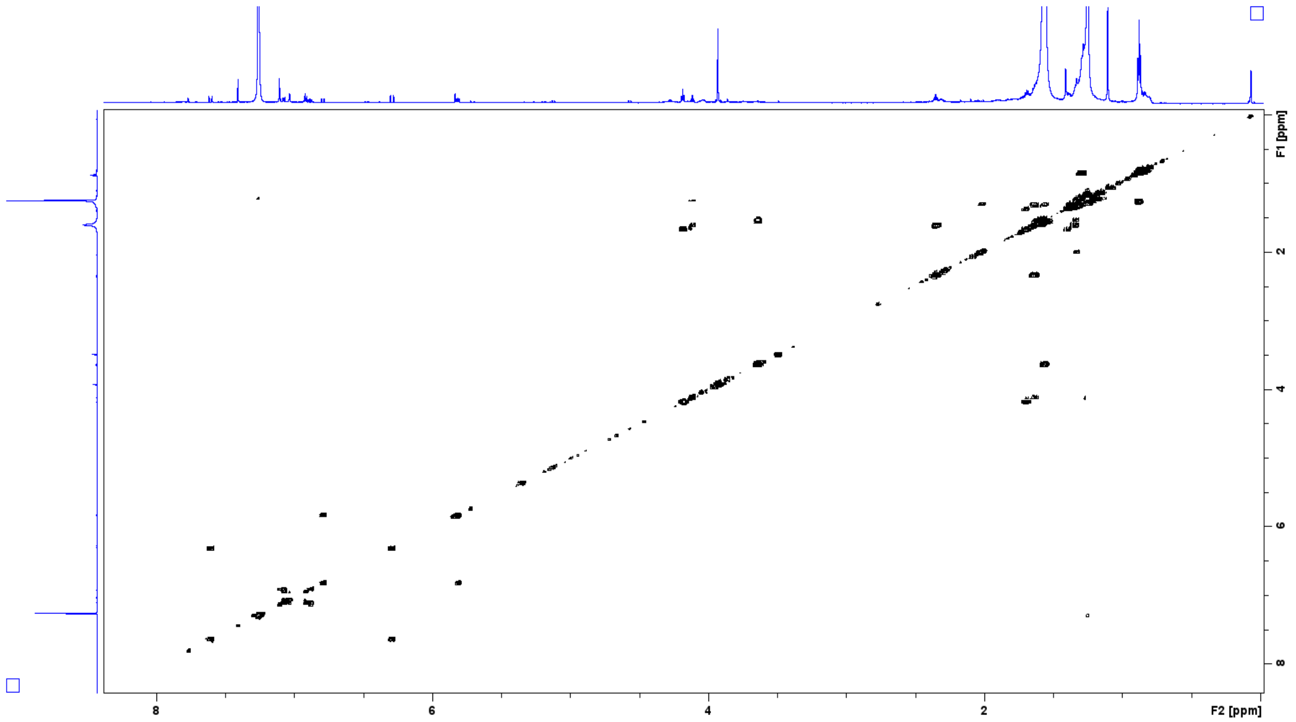

Figure S6. HSQC 2D NMR spectrum of compound **4** (700 MHz) in $CDCl_3$

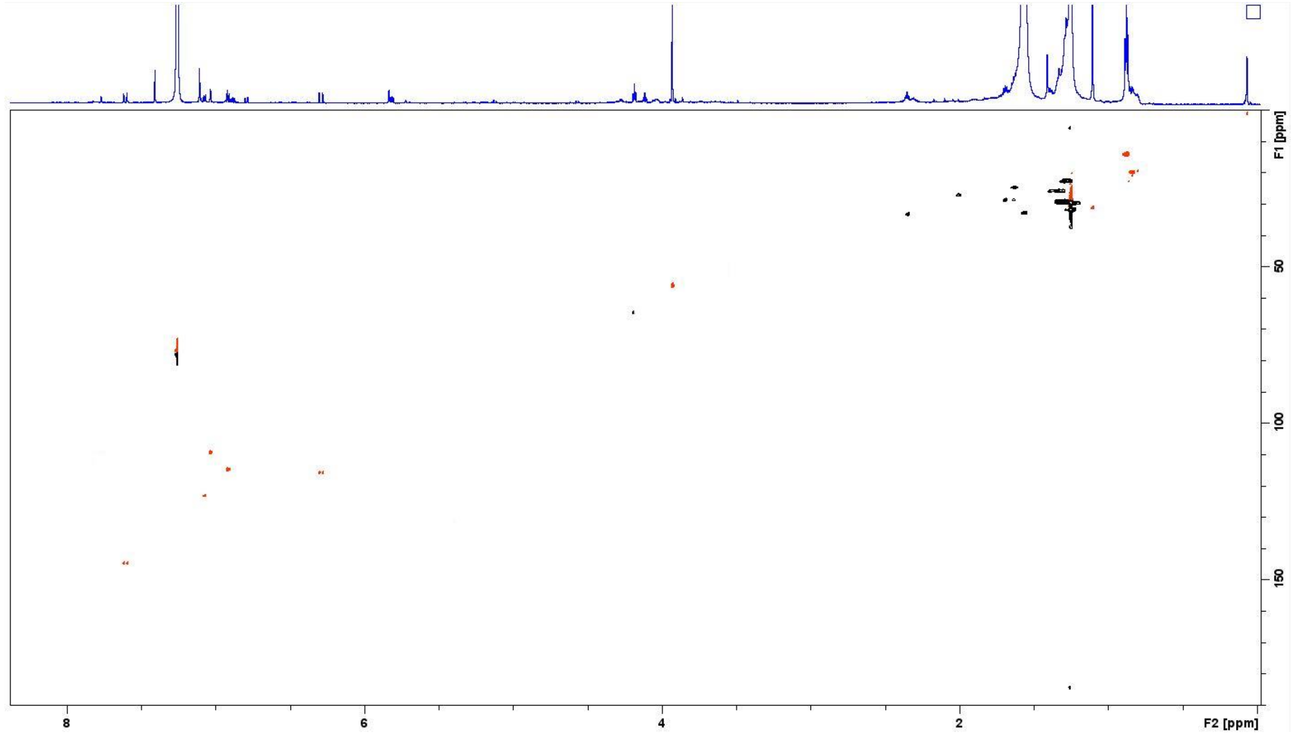

Figure S7. HMBC 2D NMR spectrum of compound **4** (700 MHz) in $CDCl_3$

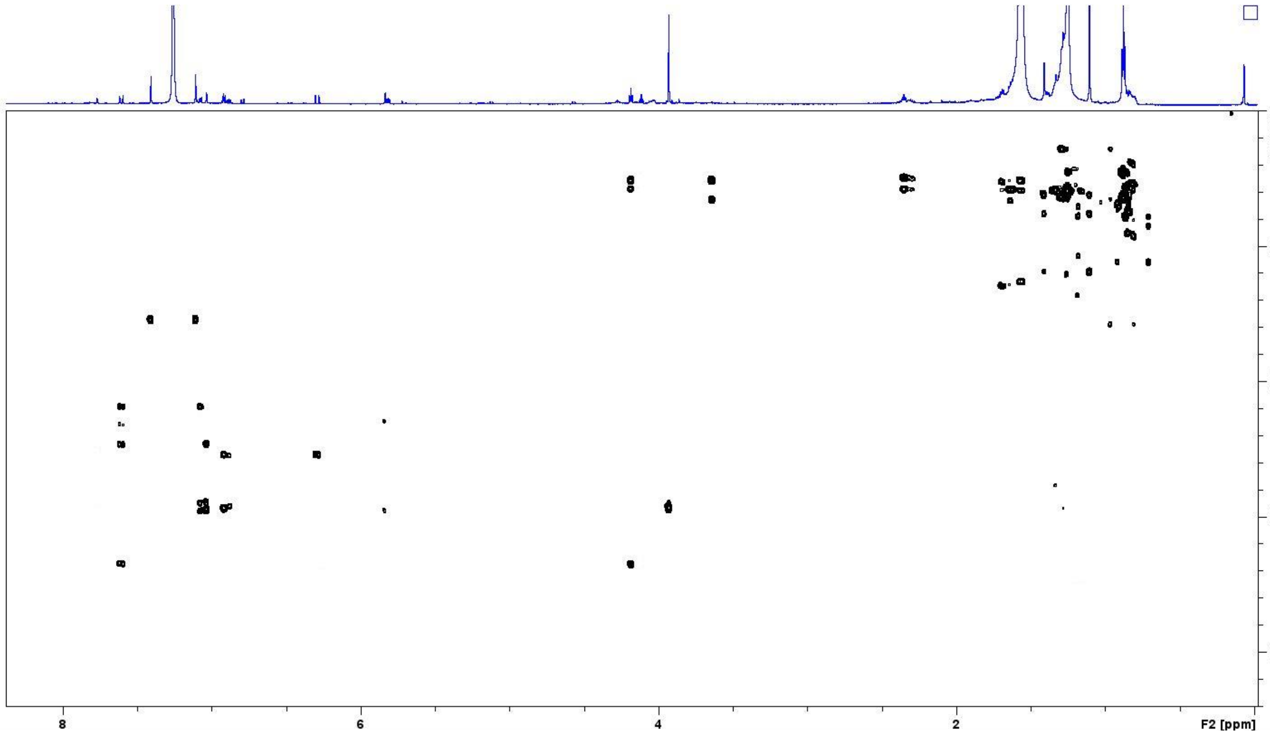

Figure S8. ^{1}H NMR spectrum of compound **5** (600 MHz) in $CDCl_3$

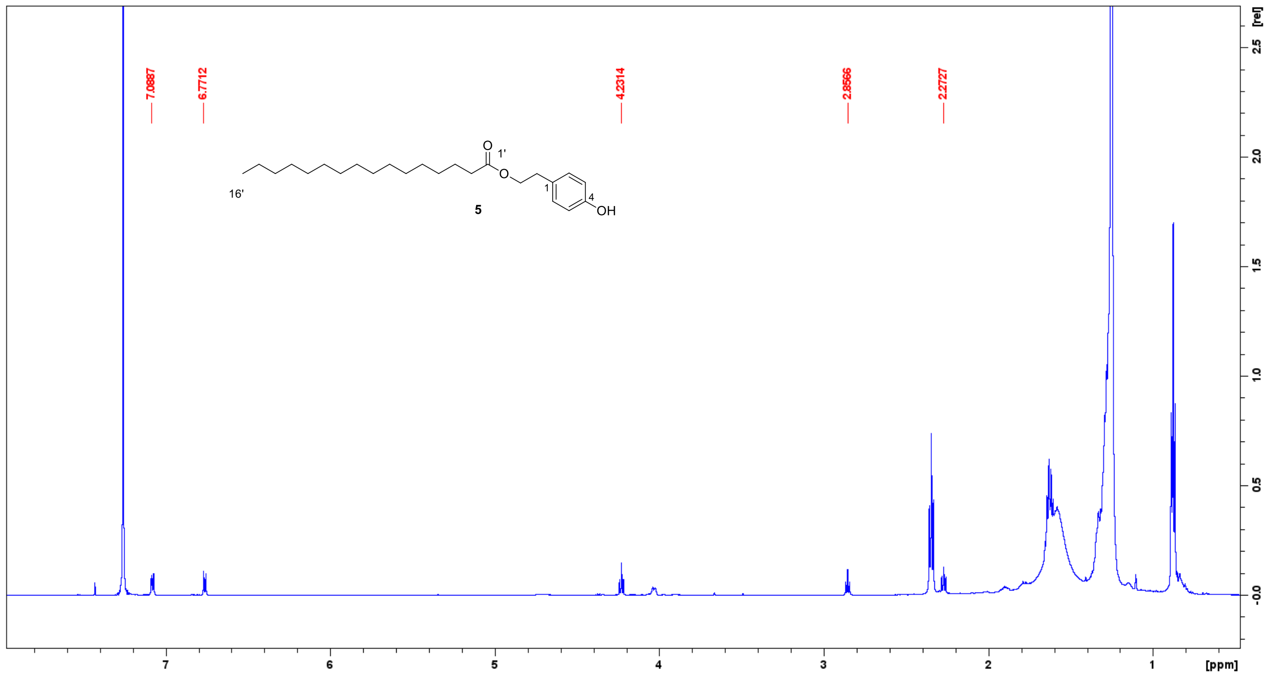

Supplement: Supplementary file 1 [file biomolecules-14-00134-s001.zip › biomolecules-2813707-supplementary.pdf]
